# Supplementary material for: Cross-sectional assessment of Angiostrongylus cantonensis transmission risk mediated by invasive apple snails in Jiangsu province of China
Source: PLoS Negl Trop Dis. 2025 Dec 2;19(12):e0013803. doi: 10.1371/journal.pntd.0013803 (PMC12688135; doi:10.1371/journal.pntd.0013803)
Supplement: S1 Text — (DOCX) [file pntd.0013803.s001.docx]

**Questionnaire on knowledge, attitudes, and practices (KAP) regarding apple snails in Jiangsu province**

Questionnaire Number: __________

Address: ________City, ________District/(City)/County, ________Sub-district/Township, ________Community/Village

**1. Basic information**

1.1 Gender: ① Male ② Female

1.2 Age: __________ years old

1.3 Ethnic Group: ___________

1.4 Education Level: ① Primary school and below ② Junior high school ③ High school or technical secondary school ④ Junior college and above

1.5 Occupation: ① Farmer ② Worker ③ Medical Worker ④ Self-employed ⑤ Student ⑥ Others (please specify: ____________)

1.6 Marital Status: ① Unmarried ② Married

**2. Knowledge on prevention and control**

**K1** Is the apple snail an invasive species?

1. Yes ② No

**K2** What color are the eggs of apple snails?

1. Pink ② White ③ Transparent

**K3** Do apple snails damage the ecological environment?

1. Yes ② No

**K4** Do apple snails harm crops?

1. Yes ② No

**K5** Can eating raw or undercooked apple snails’ meat cause parasitic infections?

① Yes ② No

**3. Attitudes toward prevention and control**

**A1** How severe do you think the health risks posed by apple snails are?

① Very severe ②No risk

**A2** If you knew that eating raw snail meat (e.g., cold snail salad) carries a risk of parasitic infection, would you still consume it?

1. Yes ② No

**A3** Do you want to learn more about the risks of apple snails?

1. Yes ② No

**A4** Would you share the knowledge about apple snails with family or others?

① Yes ② No

**4. Practices in prevention and control**

**P1** Have you ever consumed raw or undercooked snail meat?

1. Yes ② No

**P2** Have you directly handled apple snails with bare hands?

① Yes ② No

**P3** Do you regularly drink untreated water?

① Yes ② No

**P4** When eating raw fruits/vegetables, do you consume them without washing?

① Yes ② No

**P5** Do you use separate cutting boards and knives for raw and cooked foods?

1. Yes ② No

**P6** If you find apple snails, would you report it to authorities?

1. Yes ② No

**P7** Have you ever reported sightings of apple snails via the Apple Snail Inspector (ASI) app?

① Yes ② No
