# Supplementary material for: Cross-sectional assessment of Angiostrongylus cantonensis transmission risk mediated by invasive apple snails in Jiangsu province of China
Source: PLoS Negl Trop Dis. 2025 Dec 2;19(12):e0013803. doi: 10.1371/journal.pntd.0013803 (PMC12688135; doi:10.1371/journal.pntd.0013803)
Supplement: S1 Table — (DOCX) [file pntd.0013803.s002.docx]

S1 Table. Investigation on the field infection and market sales status of apple snails

| City | Field investigation | |  | Market sales investigation | |  |
| --- | --- | --- | --- | --- | --- | --- |
|  | Tested samples | Positive samples |  | Investigated markets | Existence of sales situation |  |
| Wuxi | 100 | 0 |  | 12 | 0 |  |
| Suzhou | 135 | 0 |  | 12 | 0 |  |
| Nantong | 112 | 0 |  | 12 | 0 |  |
| Yancheng | 160 | 0 |  | 14 | 0 |  |
| Taizhou | 100 | 0 |  | 12 | 0 |  |
| Suqian | 125 | 0 |  | 12 | 0 |  |
| Total | 732 | 0 |  | 74 | 0 |  |
