# Supplementary material for: Cross-sectional assessment of Angiostrongylus cantonensis transmission risk mediated by invasive apple snails in Jiangsu province of China
Source: PLoS Negl Trop Dis. 2025 Dec 2;19(12):e0013803. doi: 10.1371/journal.pntd.0013803 (PMC12688135; doi:10.1371/journal.pntd.0013803)
Supplement: Attachment — Submitted filename: PlosNTD revision 2025.pdf [file pntd.0013803.s003.pdf]

This study that investigates the circulation of A. cantonensis in apple snails in non-endemic regions of China. Please find my revision below.

Abstract

- **Line 51**: Add "health" to the phrase “gaps in public health practices.”
  Line 53: Refine the phrase “We also found that older and less-educated residents had lower awareness and poorer preventive habits.”
  I suggest "We also observed that older individuals and those with lower levels of education demonstrated lower awareness and exhibited less effective preventive habits." This revision maintains neutrality and avoids potentially stigmatizing language.

Introduction

- **Line 67**: The role of potable water in the transmission of A. cantonensis remains under investigation, as there is currently no conclusive evidence linking it to disease transmission. In the case of fruits and vegetables (primarily vegetables), contamination may result from contact with gastropod body parts, though this too remains under scrutiny.
- **Line 73**: For a more comprehensive overview of the global expansion of *A. cantonensis*, I recommend including references such as https://pubmed.ncbi.nlm.nih.gov/36343594/ https://pmc.ncbi.nlm.nih.gov/articles/PMC10077014/, as the current references (6 and 8) do not address this issue.

Results

- **Line 198**: While it is important to separate raw and cooked cutting boards to reduce the risk of cross-contamination, further clarification is needed regarding the relevance of this practice for *A. cantonensis* transmission. The potential for larvae transmission through cross-contamination in this context may be minimal or uncertain, and this should be addressed in the discussion.

Discussion

- **Line 242**: The statement ‘ecological absence does not equate to population safety’ requires further clarification. If a pathogen is absent from a region at a given time, the specific risk associated with that pathogen may not exist. The phrase could be misleading unless additional context is provided.
- **Line 251:** “The apparent absence in Jiangsu may due to limited sample size, seasonal sampling, or presently unfavorable local ecological conditions” please provide further information on these important variables

- **Line 270**: this is particularly important given the potential for rapid spread of *A. cantonensis*, especially through contaminated food sources. However, the reference cited [38] specifically discusses raw snail consumption, not waterborne transmission. Therefore, the claim about water sources should be revisited and, if applicable, supported by more relevant references.

- **General Comment**: The authors should mention the lack of specific public health guidelines, particularly in newly endemic regions of *A. cantonensis*. Interestingly, in regions where *A. cantonensis* is endemic, there is a lack of specific public health guidelines to educate the public on the risks associated with gastropod contact. For example, a recent study by [DOI: 10.1111/zph.13228] highlights the need for such guidelines in endemic areas and mention the absence of such guides.

References

- **Reference 3**: Correction needed regarding author name.
  "Carlos GT" should be corrected to "Graeff-Teixeira" to reflect the proper citation format.
