# Supplementary material for: Cross-sectional assessment of Angiostrongylus cantonensis transmission risk mediated by invasive apple snails in Jiangsu province of China
Source: PLoS Negl Trop Dis. 2025 Dec 2;19(12):e0013803. doi: 10.1371/journal.pntd.0013803 (PMC12688135; doi:10.1371/journal.pntd.0013803)
Supplement: Attachment — Submitted filename: PNTD-D-25-01764 Manuscript REVIEWED.docx [file pntd.0013803.s004.docx]

**Cross-sectional assessment of *Angiostrongylus cantonensis* transmission risk mediated by invasive apple snails in Jiangsu Province of China**

**A short title: Transmission risk of *Angiostrongylus cantonensis* via apple snails in Jiangsu, China**

Qiang Zhang^1^, Fanzhen Mao^1^, Bixian Ni^1^, Jiayao Zhang^1^, Feng Tang^1^, Xinyi Gong^1^, Yaobao Liu^1,2*^

^1^ National Health Commission Key Laboratory of Parasitic Disease Control and Prevention, Jiangsu Provincial Key Laboratory on Parasite and Vector Control Technology, Jiangsu Provincial Medical Key Laboratory, Jiangsu Institute of Parasitic Diseases Wuxi, Jiangsu 214064, China

^2^ School of Public Health, Nanjing Medical University, Nanjing, Jiangsu 211166, China

* Correspondence: Yaobao Liu: yaobao0721@163.com

# Abstract

## Background

*Angiostrongylus cantonensis*, a zoonotic nematode responsible for eosinophilic meningitis, is acquired in China principally through ingestion of raw or under-cooked invasive apple snails—the vector implicated in almost all Chinese outbreaks to date. Despite the widespread distribution of these snails in Jiangsu Province of China, systematic assessments of infection risks and public awareness remain limited.

## Methodology/Principle findings

We carried out a cross-sectional study in six prefectural-level cities in Jiangsu. In total, 732 wild apple snails were collected from different habitats and screened for *A. cantonensis* larvae by pepsin–HCl digestion and microscopy. 74 local farmers markets were inspected for the sale of live or processed apple snails. Simultaneously, a validated KAP (Knowledge, Attitudes, and Practices) questionnaire was conducted with 630 randomly selected residents. Data analysis employed SPSS for descriptive statistics and multivariate linear regression was used to identify predictors of higher scores. All wild-caught snails tested negative for *A. cantonensis*, and no apple snails were found for sale in any market. Overall, the public demonstrated a high level of knowledge and positive attitudes towards the risks posed by apple snails; however, gaps in practice were evident: 40.3% of respondents reported not separating utensils used for raw foods from those used for cooked foods, and 78.7% reported snail sightings to authorities, while only 22.1% had ever used the Apple Snail Inspector (ASI) mini-program. KAP scores differed significantly among different demographic groups. Multivariable linear regression analysis revealed that age, city of residence, and education level were significant predictors of KAP scores.

## Conclusion/Significance

While no infected snails were detected in this study, the potential risk of transmission remains because of the widespread presence of competent apple snails. Targeted health education programs that address behavioral gaps among older and less-educated residents could further minimize the threat of *A. cantonensis* introduction and safeguard both public health and ecological security.

# Author summary

In this study, we investigated the risk of human infection with the rat lungworm, *Angiostrongylus cantonensis*, which can cause severe meningitis. This parasite is often spread through invasive apple snails, which are now widespread in Jiangsu Province, China. We combined field surveys of snails with a public questionnaire to understand both ecological and behavioral risks. Although we did not find any infected snails in the wild or in local markets, our survey revealed important gaps in public practices. For example, many people do not separate utensils for raw and cooked food, and few use our reporting app for snail sightings. We also found that older and less-educated residents had lower awareness and poorer preventive habits. Our findings show that even without current detection of the parasite, the potential for outbreak remains because of widespread snails and unsafe behaviors. We recommend targeted health education, especially for high-risk groups, and continued monitoring to prevent future infections. This integrated approach will help protect both public health and local ecosystems.

# Keywords

*Angiostrongylus cantonensis*; Apple snails; Knowledge, attitude, and practice (KAP); Transmission risk.

# Introduction

*Angiostrongylus cantonensis*, the rat lungworm, first described by Chen in 1935 [1], is now recognized as the leading global cause of human eosinophilic meningitis [2]. Human infection follows ingestion of third-stage larvae (L3) residing in intermediate and paratenic hosts, or in contaminated agricultural produce, fruits, and potable water [3, 4]. After ingestion, the L3 travel from the intestines to the brain, where upon death the L3 elicit a strong immune response, leading to meningitis and severe neurological symptoms, including headaches, cranial nerve palsies, and, in severe cases, coma or death [5-7]. Originally endemic to Southeast Asia and subsequently to Pacific Islands, the parasite has expanded its range to over 30 countries across Asia, Africa, the Americas, Europe, and Australia, driven by rat migration and global trade [6, 8]. In recent years, reported neuroangiostrongyliasis cases have increased significantly in China [9-11]. Moreover, there have been multiple outbreaks, which pose a serious threat to human health and have attracted urgent public-health attention [12-15].

Human infections are driven largely by culinary habits, particularly the consumption of raw or under-cooked snail meat—fuelled by a growing appetite for exotic or "fresh" dishes. This behavior heightens exposure to L3, facilitating outbreaks of Angiostrongyliasis. In China, invasive apple snails (*Pomacea* spp.) play pivotal roles in transmitting *A. cantonensis* and almost all documented outbreaks have been traced to their ingestion [15-17]. Apple snails, now widespread across southern, southwestern, and central China, have been confirmed as a carrier in several provinces [18, 19]. Jiangsu Province is abundant in water resources and the invasive apple snail has rapidly spread in this area, causing severe damage to aquatic ecosystems including farmlands and ponds in recent years [20]. It has been listed as one of the eight Level 1 controlled species in Jiangsu province's first catalog of invasive alien species [21]. To date, over 1,200 infestation sites have been documented by our real-time monitoring platform—Apple Snail Inspector (ASI) across various aquatic environments including farmlands, park wetlands, and riverine habitats [22]. Although no autochthonous human Angiostrongyliasis cases have yet been reported in Jiangsu, the confluence of widespread vector presence and risky food behaviors makes sustained monitoring and targeted health education an urgent public-health priority.

A recent survey has shown that approximately 27% of residents were unable to identify apple snails in Shanghai [23], and an online survey revealed that 10.6% of respondents would still knowingly eat raw food, despite the risk of parasitic infection [24]. These gaps between recognition and practice underscore the critical importance of foodborne disease prevention during the annual reproductive peak. The Knowledge, attitudes, and practices (KAP) model is a foundational tool in public health research. It offers a systematic approach to understanding health behaviors, empowering researchers to design impactful interventions and assess their success. KAP surveys play a vital role in this process by assessing the current level of knowledge within a population regarding specific health issues [25]. By quantitatively analyzing public knowledge, preventive attitudes, and behavioral practices regarding apple snails, cognitive vulnerabilities can be precisely identified. Sustained KAP monitoring not only evaluates the effectiveness of awareness campaigns but also guides the establishment of a community-based "identification-prevention-reporting" through behavior change theories, ultimately forming a closed-loop control system [26].

Therefore, this study aimed to evaluate the infection status of *A. cantonensis* in invasive apple snails in Jiangsu Province, and simultaneously investigate the KAP of local residents regarding apple snails and *A. cantonensis* transmission. Findings will be translated into precision interventions: risk-communication campaigns for low-awareness groups, enhanced alerts for those underestimating danger, and behavior-nudging measures to correct unsafe practices. These integrated efforts should effectively reduce the infection risk of *A. cantonensis*, protect public health and ecological security.

# Methods

## Field sampling and detection of apple snails

Six prefectural-level cities in Jiangsu (Wuxi, Suzhou, Nantong, Yancheng, Taizhou, and Suqian) were selected to cover the province’s major drainage basins and to coincide with ASI hot-spot data. ASI is a smartphone-based, crowd-sourced surveillance system developed by our institute for real-time reporting and monitoring of invasive apple snails [22]. The platform consists of a WeChat mini-program accessible to the public. Members of the public can report snail sightings by uploading geotagged photographs and information on the snail's habitat through the mini-program. These reports are instantly submitted to a centralized database managed by our research team. A minimum of four sampling sites per city were selected. However, in cities with fewer than four documented infestation sites, all available sites were enrolled, prioritizing locations with both apple snail infestations and signs of rodent activity. Snails were collected between June and September 2024 (the species’ peak reproductive window) using a combination of dip-netting and gloved hand-picking. At least 25 apple snails were collected per site, with a total sample size reaching ≥100 snails for subsequent detection. Collected snails were transported alive to the laboratory and identified morphologically as apple snails. To detect *A. cantonensis* infection, two standard parasitological techniques were employed: lung tissue microscopy and artificial digestion with pepsin-HCl [27]. Briefly, snails were euthanized by freezing at -20℃ for 24 hours, followed by necropsy to microscopically examine lung tissue for larvae. The entire soft tissue of each snail was then digested in 1% pepsin in 0.7% HCl at 37℃ for 2-3 hours with constant agitation. After digestion, the mixture was filtered through a 30-mesh sieve, and the sediment was examined under a microscope for L3 larvae of *Angiostrongylus cantonensis*.

During the monitoring period, field surveys were carried out at a minimum of three markets per site each month. If apple snails had been found on sale at these markets, specimens would have been purchased and subjected to laboratory detection. Unfortunately, none was found.

## Population-based KAP survey

We developed a 16-item KAP questionnaire tailored to the Jiangsu context (S1 Text). Questionnaire content validity was refined through a group meeting with experts in parasitology, epidemiology, health education, and veterinary public health. The questionnaire comprised three dimensions: (1) Knowledge: 5 items assessing recognition of apple snails, awareness of their health risks, and understanding of preventive measures (correct answer scored 1 and incorrect answer scored 0); (2) Attitude: 4 items evaluating risk perception and willingness to adopt protective behaviors (positive attitude scored 1 and negative attitude scored 0); (3) Practice: 7 items measuring self-reported behaviors related to snail handling, consumption, and reporting (good practice scored 1 and poor practice scored 0). The internal consistency reliability of the KAP questionnaire was assessed using Cronbach's alpha coefficient, a measure of how closely related a set of items are as a group (ranging from 0 to 1, with higher values indicating greater internal consistency). The tool demonstrated acceptable reliability (Cronbach's α = 0.63) and was validated through pilot testing. The surveys were implemented in communities and schools adjacent to their infestation sites, with a requirement of surveying ≥ 100 respondents per monitoring site. Demographic variables included city of residence, age, gender, education, occupation, and marital status.

## Statistical analysis

Data were reported using a customized Epidata database [28] and entered using a dual-entry verification method by two independent operators. All visualizations were produced using Microsoft Excel 2016. Statistical analyses were performed with SPSS 23.0. Descriptive statistics (mean ± standard deviation, x̄ ± s) summarized KAP scores across subgroups. Intergroup differences were evaluated via independent t-tests and one-way ANOVA. Multivariate linear regression identified predictors of KAP scores, adjusting for covariates such as city of residence, age, education, and occupation. Statistical significance was set at *P* < 0.05 (two-tailed).

## Ethical considerations

This study was reviewed and approved by the Institutional Ethics Committee of Jiangsu Institute of Parasite Diseases. Written informed consent was obtained from all participants, and data were anonymized to ensure confidentiality.

# Results

## No infected apple snails and market sales observed

In total, 732 wild apple snails were collected across the six surveyed cities in Jiangsu Province (S1 Table): Wuxi (100), Suzhou (135); Nantong(112), Yancheng (160), Taizhou (100), Suqian (125). Laboratory detection revealed no *A. cantonensis* larvae in any specimen. Concurrently, market inspections across 74 local markets (Wuxi: 12; Suzhou: 12; Nantong: 12; Yancheng: 14; Taizhou: 12; Suqian: 12) found no apple snails offered for sale, indicating effective market regulation and a low risk of human exposure to infected snails in these regions.

**S1 Table. Investigation on the field infection and market sales status of apple snails**

| City | Field investigation | |  | Market sales investigation | |  |
| --- | --- | --- | --- | --- | --- | --- |
|  | Tested samples | Positive samples |  | Investigated markets | Existence of sales situation |  |
| Wuxi | 100 | 0 |  | 12 | 0 |  |
| Suzhou | 135 | 0 |  | 12 | 0 |  |
| Nantong | 112 | 0 |  | 12 | 0 |  |
| Yancheng | 160 | 0 |  | 14 | 0 |  |
| Taizhou | 100 | 0 |  | 12 | 0 |  |
| Suqian | 125 | 0 |  | 12 | 0 |  |
| Total | 732 | 0 |  | 74 | 0 |  |

## Responses of the surveyed population to the KAP questions regarding apple snails

In total, 630 questionnaires were collected in this study. In the knowledge component, the percentage of correct answers was relatively high (＞80% correct on all items), indicating that the public has a certain level of understanding in these areas. Regarding attitudes, most respondents showed a positive attitude with 98.3% of respondents expressing a desire to learn about apple snail hazards, and 97.3% willing to share relevant knowledge with others. In terms of practices, although a certain number of people separate cutting boards and knives for raw and cooked food, there is still a significant portion who do not (40.3%). Moreover, only 78.7% of respondents would report the discovery of apple snails to the authorities and just 22.1% had ever used the ASI platform (Fig 1)

**Fig 1. Responses of the surveyed population to the knowledge, attitudes, and practices (KAP) questions about apple snails**

## KAP scores across demographic subgroups

KAP scores showed significant disparities among different demographic groups (Table 1). Univariate analysis revealed that scores varied significantly by city of residence, age, education level, occupation, and marital status. In contrast, no significant difference was found between genders. By city (*P* < 0.001), Nantong exhibited the highest score (14.10 ± 1.92), while Suqian had the lowest score (12.44 ± 2.20). Gender differences were negligible (males 13.35 ± 1.90 vs. females 13.25 ± 2.15; *P* = 0.549). Age emerged as a critical factor: individuals aged <20 scored highest (13.82 ± 1.80), while those >40 scored lowest (12.93 ± 2.08) (*P* < 0.001). Education level also significantly influenced scores, with higher education (e.g., college graduates: 13.91 ± 1.84) associated with better KAP scores compared to primary education (13.11 ± 2.09) (*P* < 0.001). Occupation-based analysis highlighted students (13.83 ± 1.77) and “other” occupations (13.84 ± 1.49) as high-performing groups, whereas farmers scored lowest (12.59 ± 2.12) (*P* < 0.001). Marital status further stratified results, with unmarried individuals (13.75 ± 1.90) outperforming married participants (13.12 ± 2.04) (*P* < 0.001).

**Table 1. Comparison of KAP scores in each dimension with different demographic characteristic**

| Characteristics | Number of surveys | Knowledge  (x̄ ± s) | Attitude  (x̄ ± s) | Practice  (x̄ ± s) | Total  (x̄ ± s) |
| --- | --- | --- | --- | --- | --- |
| City |  |  |  |  |  |
| Wuxi | 100 | 4.51±1.02 | 3.84±0.37 | 5.47±1.03 | 13.82±2.07 |
| Suzhou | 100 | 4.45±1.10 | 3.74±0.51 | 5.47±1.05 | 13.66±1.99 |
| Nantong | 105 | 4.43±1.28 | 3.88±0.36 | 5.80±0.58 | 14.10±1.92 |
| Yancheng | 116 | 4.94±0.30 | 3.87±0.45 | 4.86±1.05 | 13.67±1.25 |
| Taizhou | 100 | 3.38±1.18 | 3.24±0.57 | 5.50±1.16 | 12.12±1.81 |
| Suqian | 109 | 4.15±1.31 | 3.16±0.72 | 5.14±1.11 | 12.44±2.20 |
| *F* |  | 24.488 | 44.494 | 11.471 | 19.330 |
| *P* |  | <0.001^*^ | <0.001^*^ | <0.001^*^ | <0.001^*^ |
| Gender |  |  |  |  |  |
| Male | 339 | 4.39±1.09 | 3.60±0.62 | 5.36±1.03 | 13.35±1.90 |
| Female | 291 | 4.24±1.27 | 3.65±0.57 | 5.36±1.09 | 13.25±2.15 |
| *t* |  | 1.562 | -1.202 | 0.065 | 0.599 |
| *P* |  | 0.119 | 0.230 | 0.949 | 0.549 |
| Age |  |  |  |  |  |
| ＜20 | 152 | 4.77±0.90 | 3.73±0.59 | 5.32±0.95 | 13.82±1.80 |
| 20-40 | 135 | 4.47±1.02 | 3.61±0.53 | 5.60±0.96 | 13.68±1.92 |
| ＞40 | 343 | 4.07±1.27 | 3.58±0.62 | 5.28±1.12 | 12.93±2.08 |
| *F* |  | 21.522 | 3.440 | 4.565 | 13.793 |
| *P* |  | <0.001^*^ | 0.033^*^ | 0.011^*^ | <0.001^*^ |
| Education level |  |  |  |  |  |
| Primary school and below | 230 | 4.27±1.29 | 3.61±0.65 | 5.22±1.05 | 13.11±2.09 |
| Junior high school | 150 | 4.09±1.29 | 3.55±0.62 | 5.10±1.10 | 12.74±2.09 |
| High school or technical secondary school | 99 | 4.42±0.91 | 3.67±0.54 | 5.61±0.99 | 13.70±1.68 |
| Junior college and above | 151 | 4.56±0.97 | 3.68±0.51 | 5.67±0.95 | 13.91±1.84 |
| *F* |  | 4.452 | 1.527 | 10.975 | 10.960 |
| *P* |  | 0.004^*^ | 0.206 | <0.001^*^ | <0.001^*^ |
| Occupation |  |  |  |  |  |
| Farmer | 149 | 3.95±1.37 | 3.55±0.63 | 5.09±1.19 | 12.59±2.12 |
| Worker | 117 | 4.20±1.18 | 3.63±0.60 | 5.32±0.99 | 13.15±1.96 |
| Medical Worker | 58 | 4.34±1.15 | 3.57±0.62 | 5.55±1.13 | 13.47±2.20 |
| Self-employed | 64 | 4.22±1.24 | 3.59±0.61 | 5.33±1.156 | 13.14±2.38 |
| Student | 157 | 4.78±0.89 | 3.71±0.59 | 5.34±0.94 | 13.83±1.77 |
| Others | 85 | 4.39±0.95 | 3.62±0.49 | 5.82±0.76 | 13.84±1.49 |
| *F* |  | 8.663 | 1.308 | 5.850 | 7.706±2.02 |
| *P* |  | <0.001^*^ | 0.259 | <0.001^*^ | <0.001^*^ |
| Marital Status |  |  |  |  |  |
| Unmarried | 186 | 4.69±1.00 | 3.70±.59 | 5.36±0.97 | 13.75±1.90 |
| Married | 444 | 4.17±1.21 | 3.59±.59 | 5.36±1.09 | 13.12±2.04 |
| *t* |  | 5.542 | 2.253 | -0.002 | 3.626 |
| *P* |  | <0.001^*^ | 0.025^*^ | 0.999 | <0.001^*^ |

^*^Statistically significant.

## Multivariate predictors of KAP scores

Variables with a univariate *p*-value of total scores below 0.05 were further analyzed using multivariate linear regression analysis to identify key predictors of KAP scores. Three factors remained significant predictors of total KAP score: city of residence (all cities scored higher than the Suqian reference group. Suqian was chosen as the reference category for the city variable as it demonstrated the lowest mean KAP scores), younger age and higher education level (Table 2), whereas occupation and marital status did not independently predict scores in the adjusted model.

**Table 2. Multivariate linear regression analysis of the influence of different characteristics on KAP scores**

| Characteristics | *β* | Standard error | Standardized  *β* | *P* |
| --- | --- | --- | --- | --- |
| Age | -0.030 | 0.010 | -0.307 | 0.002^*^ |
| Marital Status (Ref = Married) | -0.461 | 0.372 | -0.104 | 0.216 |
| City (Ref = Suqian) |  |  |  |  |
| Wuxi | 1.214 | 0.275 | 0.220 | <0.001^*^ |
| Suzhou | 1.265 | 0.267 | 0.229 | <0.001^*^ |
| Nantong | 2.025 | 0.259 | 0.374 | <0.001^*^ |
| Yancheng | 1.683 | 0.263 | 0.323 | <0.001^*^ |
| Taizhou | 0.609 | 0.286 | 0.110 | 0.034 |
| Education level (Ref = Primary School and Below) |  |  |  |  |
| Junior high school | 0.484 | 0.226 | 0.102 | 0.033^*^ |
| High school or technical secondary school | 1.342 | 0.288 | 0.242 | <0.001^*^ |
| College and above | 1.274 | 0.333 | 0.270 | <0.001^*^ |
| Occupation (Ref = Farmer) |  |  |  |  |
| Worker | -0.494 | 0.292 | -0.095 | 0.091 |
| Medical Worker | -0.702 | 0.377 | -0.101 | 0.063 |
| Self-employed | -0.329 | 0.331 | -0.049 | 0.322 |
| Student | 0.057 | 0.538 | 0.012 | 0.915 |
| Others | 0.071 | 0.328 | 0.012 | 0.829 |

^*^Statistically significant.

# Discussion

The present findings provide a critical assessment of the current status of *A. cantonensis* infection risk mediated by invasive apple snails in Jiangsu Province of China, and integrates snail surveillance with a detailed analysis of human behavior (KAP). Although no infected snails or market sales were detected, the pronounced practice gaps revealed by the KAP survey demonstrate that ecological absence does not equate to population safety. The integration of field data and behavioral insights provides policymakers with an evidence-based dual perspective on both ecological and behavioral dimensions to design targeted interventions and strengthen health education.

Although invasive apple snails are a significant vector for *A. cantonensis* [29], none of 732 wild specimens collected across six cities in Jiangsu carried the parasite. However, the ecological adaptability of apple snails and their widespread distribution in Jiangsu’s water networks necessitate vigilance [22, 30]. Multiple provinces adjacent to Jiangsu have documented infections of *A. cantonensis* in apple snails [31, 32]. The apparent absence in Jiangsu may be due to limited sample size, seasonal sampling, or presently unfavorable local ecological conditions [33]. Additionally, no apple snail sales were found in the 74 surveyed local markets, indicating effective market regulation in these regions, which likely reduces the direct risk of human infection through the consumption of infected snails. However, the morphological overlap between apple snails and *Cipangopaludina chinensis* [34] raises the possibility of undetected misidentification during the market surveys. Therefore, continuous surveillance, as recommended by the World Health Organization for neglected tropical diseases, is essential to detect early-stage zoonotic shifts [35].

The KAP survey results show that the residents in Jiangsu have a relatively high knowledge level of apple snails. This could be attributed to previous health education efforts and increased public awareness of food-borne diseases [25, 36]. However, a small proportion of the population remains unaware of apple snails and their associated risk. In terms of attitude, most respondents had a positive attitude, such as a strong willingness to learn about apple snail hazards and disseminate health information, providing a receptive basis for future interventions. However, previous research has shown that knowledge does not reliably translate into preventive behaviors, especially when there is a lack of perceived personal risk or motivation [37]. Although a certain number of households separated cutting boards and knives used to prepare raw and cooked items, 40.3% did not, creating a clear pathway for cross-contamination. This is particularly important given the potential for rapid spread of *A. cantonensis* through contaminated food and water sources [38]. Moreover, the low reporting rates of apple snail discoveries (78.7% to authorities and 22.1% using ASI) underscore the need for improvement in public participation in surveillance.

Demographic disparities in KAP scores were pronounced in this study. Respondents aged < 20, those with a higher level of education, students, and unmarried individuals generally had higher scores, perhaps because younger age groups access diverse information channels, and higher education fosters stronger information-seeking and risk-perception skills. In contrast, farmers had the lowest scores, which could be related to their limited access to health education resources and different living and working environments. This is consistent with previous research indicating that education level is a robust determinant of health knowledge and behavior [25, 39]. These results highlight the need for a differentiated health education strategy. For example, concise, actionable training modules and pictorial materials should be designed specifically for farmers, delivered through village health workers to maximize reach and impact.

Multivariate regression analysis identified age, city of residence, and education level as the significant predictors of KAP scores. Furthermore, the significant differences in KAP scores across cities highlight the need for region-specific strategies. Cities with higher KAP scores such as Nantong, may benefit from reinforcing existing educational programs, while cities with lower scores like Suqian, require more intensive interventions. This approach aligns with the principles of community-based participatory research [40], which emphasizes the importance of local context in designing effective public health initiatives. Additionally, promoting education-based prevention measures, such as incorporating apple snail related knowledge into school curricula and community education programs [41, 42], could offer a scalable, education-centered pathway to raise public awareness and convert knowledge into sustained preventive behaviors.

This study has several limitations. First, the cross-sectional design precludes causal inferences between KAP factors and infection risks. Second the sample size is insufficient and the sampling focused on urban and peri-urban areas; rural regions with higher agricultural activity may face distinct challenges. Third, because all specimens were screened solely by pepsin–HCl digestion and microscopy, infections with very low larval burdens may have been missed. Finally, it is important to emphasize that this survey targeted only the invasive apple snail, a recognized intermediate host; failure to detect *A. cantonensis* in this species does not imply its absence from other local intermediate or paratenic hosts.

Therefore, longitudinal studies are essential to monitor behavioral changes following interventions, and future research must extend to broader geographical regions (especially rural and remote areas) to enable a comprehensive assessment of both ecological and behavioral risks. Meanwhile, deploying molecular tools such as conventional PCR, qPCR or LAMP in future surveys will improve detection sensitivity and allow more precise monitoring of the parasite’s transmission dynamics. Additionally, future surveillance should be broadened to include a wider range of potential hosts to provide a more comprehensive assessment of the local transmission risk and to understand the role of different species in sustaining the parasite's life cycle.

# Conclusion

This study highlights the importance of public awareness and behavior in preventing *A. cantonensis* infection. Although the parasite was not detected in Jiangsu Province in this survey, the persistent gaps in public knowledge and practices underscore the need for sustained efforts to improve food safety and disease prevention. A sustained, integrated strategy combining real-time surveillance, targeted education tailored to high-risk population, and evidence-based policy measures could safeguard public health in an era of increasing zoonotic threats.

# Abbreviations

*A. cantonensis*: *Angiostrongylus cantonensis*

KAP: Knowledge, attitudes, and practices

L3: Third-stage larvae

ASI: Apple snail inspector

# Acknowledgements

Not applicable.

# Supporting information

**S1 Text: Questionnaire on knowledge, attitudes, and practices (KAP) regarding apple snails in Jiangsu province**

**S1 Table: Investigation on the field infection and market sales status of apple snails**

# Author contributions

**Conceptualization:** Qiang Zhang, Fanzhen Mao, Bixian Ni, Jiayao Zhang, Feng Tang, Xinyi Gong, Yaobao Liu

**Data curation:** Qiang Zhang, Jiayao Zhang,

**Formal analysis:** Qiang Zhang

**Funding acquisition:** Qiang Zhang, Yaobao Liu

**Investigation:** Qiang Zhang, Fanzhen Mao, Bixian Ni, Jiayao Zhang, Feng Tang, Xinyi Gong, Yaobao Liu

**Methodology:** Qiang Zhang, Yaobao Liu

**Project administration:** Qiang Zhang, Fanzhen Mao, Bixian Ni, Jiayao Zhang, Feng Tang, Xinyi Gong, Yaobao Liu

**Resources:** Qiang Zhang, Fanzhen Mao, Bixian Ni, Jiayao Zhang, Feng Tang, Xinyi Gong, Yaobao Liu

**Supervision:** Qiang Zhang, Yaobao Liu

**Validation:** Qiang Zhang, Yaobao Liu

**Visualization:** Qiang Zhang, Yaobao Liu

**Writing–original draft:** Qiang Zhang

**Writing–review & editing:** Yaobao Liu

# Reference

1.	Chen XT. Un nouveau nématode pulmonaire, *Pulmonema cantonensis*, n. g., n. sp. des rats de Canton. Ann Parasitol Hum Comp. 1935;13:312-7.

2.	Wang QP, Lai DH, Zhu XQ, Chen XG, Lun ZR. Human angiostrongyliasis. The Lancet Infect Dis, 2008;8:621-30.

3.	Graeff-Teixeira C, da Silva ACA, Yoshimura K. Update on eosinophilic meningoencephalitis and its clinical relevance. Clin Microbiol Rev, 2009;22:322-48.

4.	Cowie RH. Biology, systematics, life cycle, and distribution of *Angiostrongylus cantonensis*, the cause of rat lungworm disease. Hawaii J Med Public Health, 2013;72(6) Suppl 2:6-9.

5.	Wang QP, Wu ZD, Wei J, Owen RL, Lun ZR. Human *Angiostrongylus cantonensis*: an update. Eur J Clin Microbiol Infect Dis. 2012;31:389-95.

6.	Barratt JL, Ellis J. *Angiostrongylus cantonensis*: a review of its distribution, molecular biology and clinical significance as a human pathogen- CORRIGENDUM. Parasitology. 2019;146:1360.

7.	Cheng DH, Jiang TG, Zeng WB, Li TM, Jing YD, Li ZQ, et al. Identification and co-regulation pattern analysis of long noncoding RNAs in the mouse brain after *Angiostrongylus cantonensis* infection. Parasite Vector. 2024;17:205.

8.	Huang JL, Wang Y, Zhou X. Status and control of common food-borne parasitic diseases in China: a review. Chin J Schisto Control. 2021;33:424-9.

9.	Luo JW, Tian HC, Li CY, Zhang LP, Xie H, Tie L, et al. Investigation on the first found local cases of angiostrongyliasis cantonensis in Sichuan Province. J Prev Med Inf. 2022;38:635-7.

10.	Liu YT, Li GX. Severe angiostrongyliasis in a child. Chin J Parasitol Parasit Dis. 2022;40:813-6.

11.	Yao SH, Wu YC, Li QC, Liu P. Case analysis and prevalence of angiostrongyliasis cantonensis in Dali administrative distract from 2007 to 2013. J Trop Med. 2015;15:1413-7.

12.	Chen F, Chen SR, Li KR, Li TH, Fang W, Luo JJ. Investigation on outbreak of angiostrongyliasis cantonensis due to consumption of snail food in Dali City. Chin J Parasitol Parasit Dis. 2011;23:687-90.

13.	Lin JX, Li YS, Zhu K, Chen BJ, Cheng YZ, Lin JC, Cao Y, Chen RZ. Epidemiological study on group infection of *Angiostrongylus cantonensis* in Changle city. Chin J Parasitol Parasit Dis. 2003;02:48-50.

14.	Li TM, Chen F, Fang W, Chen SR, Li T, Li KR, et al. Analysis of surveillance cases of angiostrongyliasis in Dali from 2010 to 2016. J Trop Dis Parasitol. 2017;15:167-9.

15.	Zheng XY, Yin CH, Guo ZZ, Qi HY, Li XL, Diao ZL, et al: Epidemiological analysis on 141 cases of *Angiostrongyliasis cantonensis* in Beijing. Chin J Epidemiol. 2008;29:27-9.

16.	McAuliffe L, Fortin Ensign S, Larson D, Bavaro M, Yetto J, Cathey M, et al. Severe CNS angiostrongyliasis in a young marine: a case report and literature review. Lancet Infect Dis. 2019;19:e132-e42.

17.	Tong ZJ, Hu XM, Li CY, Fu FM, Wang GY, Yan S. A retrospective investigation of the *Angiostrongyliasis cantonensis* in Hainan Province. Chin Trop Med. 2013;13:351-2+5.

18.	Xie BB, Liu C, Hou ZY, Wu XN, Sun QM, Li TM, et al. Infection status of intermediate host snails of *Angiostrongylus cantonensis* in three regions of Hainan Province in 2022. Parasites and Infectious Diseases. 2023;21:239-42+48.

19.	Zhang J, Li Y, Zhu AY, Yao DC. A meta-analysis of prevalence of *Angiostrongylus cantonensis* infection among snail intermediate host in China from 2010 to 2021. J Prev Med Inf. 2022;38:1617-25.

20.	Wei R, Xie HF, Hong F, Gong WR, Du YZ. Risk analysis of the alien invasive pest Pomace [sic] spp. in Jiangsu province. J Yangzhou Univ (Agric Life Sci Ed). 2021;42:119–24+37.

21.	Announcement on the release of the first list of invasive alien species in Jiangsu province. In: Jiangsu provincial department of ecology and environment, Jiangsu provincial department of agriculture and rural affairs. 2022. http://sthjt.jiangsu.gov.cn/art/2022/5/20/art_83558_10456180.html.

22.	Zhang Q, Ding X, Zhang Y, Yang Y, Mao F, Ni B, et al. A smartphone-based crowd-sourced real-time surveillance platform (apple snail inspector) for the invasive snails: a design and development study. Parasite vector. 2024;17:78.

23.	Wang JR, Lu Q, Lu XC, Yu SY, Liu HZ. A study on the risk of *Pomacea canaliculate* and *Angiostrongylus cantonensis* in Pudong New Area, Shanghai. Chin J Hyg lnsect & Equip. 2025;31:1-4.

24.	Ni B, Zhang Q, Tang F, Zhang J, Mao F, Liu Y. Risk factors of foodborne parasitic diseases still remain in China: A WeChat-based knowledge, attitudes, and practices study. BMC Public Health. 2024;24:2763.

25.	Zarei F, Dehghani A, Ratansiri A, Ghaffari M, Raina SK, Halimi A, et al. ChecKAP: A checklist for reporting a knowledge, attitude, and practice (KAP) study. Asian Pac J Cancer P. 2024;25:2573-7.

26.	Michie S, van Stralen MM, West R. The behaviour change wheel: a new method for characterising and designing behaviour change interventions. Implement Sci. 2011;6:42.

27.	Cai WW, Lin CX, Zheng D, Xie HG. A comparative study of lung-microscopy and tissue homogenate in detecting *Angiostrongylus cantonensis* in the *Pomacea canaliculata*. Chin Trop Med. 2022;22:964-8.

28.	EpiData Association. EpiData Software. Odense, Denmark. https://www.epidata.dk.

29.	Lv S, Zhang Y, Liu HXtEXT ON THE SINGLE FIGURE COULD BE LARGER FOR READABILITY, Hu L, Yang K, Steinmann P, et al. Invasive snails and an emerging infectious disease: results from the first national survey on *Angiostrongylus cantonensis* in China. PLoS Negl Trop Dis. 2009;3:e368.

30.	Yang QQ, Liu SW, He C, Yu XP. Distribution and the origin of invasive apple snails, *Pomacea canaliculata* and *P. maculata* (Gastropoda: Ampullariidae) in China. Sci Rep. 2018;8:1185.

31.	Fan ZH, Zhan T, Sun CS, Xiang KX, Jiang XY, Wang WC, et al. First detection of *Angiostronylus cantonensis* infection in *Pomacea canaliculata* in Ma’anshan City. J Trop Dis Parasitol. 2024;22:364-6+74.

32.	Zheng D, Cai WW, Jiang DW, Lin CX, Xie HG, Chen ZY, et al. Investigation on the infection of *Angiostrongylus cantonensis* in snails in public greenbelts of Fuzhou. Chinese Journal of Food Hygiene. 2024;36:1161-5.

33.	Jarvi S, Prociv P. *Angiostrongylus cantonensis* and neuroangiostrongyliasis (rat lungworm disease): 2020. Parasitology. 2021;148:129-32.

34.	Ye M, Fan TQ, Chen L, Chen Y, Zhu SL, Wu J, et al. Comparative study on morphology of radula of *Pomacea canaliculata*, *P. maculata* and *Cipangopaludina chinensis*. Chinese Journal of Zoology. 2017;52:97-107.

35.	Jesudason T. Global progress report on neglected tropical diseases. Lancet Infect Dis. 2024;24:e420.

36.	Wang Y, Huang YX, Liu YB. Effect of WeChat public account on health science popularization of parasitic diseases. J Trop Dis Parasitol. 2021;19 01:52-5.

37.	Conner M, Norman P. Understanding the intention-behavior gap: The role of intention strength. Front Psychol. 2022;13:923464.

38.	Tsai H, Liu Y, Kunin C, Lee S, Chen Y, Lin H, et al. Eosinophilic meningitis caused by *Angiostrongylus cantonensis*: report of 17 cases from Taiwan. Am J Med. 2021;111:109-14.

39.	Baker DW, Wolf MS, Feinglass J, Thompson JA, Gazmararian JA, Huang J. Health literacy and mortality among elderly persons. Arch Int Med. 2007;167:1503-9.

40.	Wallerstein N, Duran B. Community-based participatory research contributions to intervention research: the intersection of science and practice to improve health equity. Am J Public Health. 2010;100 Suppl 1:S40-6.

41.	Berkman ND, Sheridan SL, Donahue KE, Halpern DJ, Crotty K. Low health literacy and health outcomes: an updated systematic review. Ann Intern Med. 2011;155:97-107.

42.	Questa K, Das M, King R, Everitt M, Rassi C, Cartwright C, et al. Community engagement interventions for communicable disease control in low- and lower- middle-income countries: evidence from a review of systematic reviews. Int J Equity Health. 2020;19:51.
