# Supplementary material for: Cross-sectional assessment of Angiostrongylus cantonensis transmission risk mediated by invasive apple snails in Jiangsu province of China
Source: PLoS Negl Trop Dis. 2025 Dec 2;19(12):e0013803. doi: 10.1371/journal.pntd.0013803 (PMC12688135; doi:10.1371/journal.pntd.0013803)
Supplement: Attachment — Submitted filename: Response to Reviewers.docx [file pntd.0013803.s006.docx]

26^th^ November 2025

Dear editor,

Submission ID: PNTD-D-25-01764

“Cross-sectional assessment of *Angiostrongylus cantonensis* transmission risk mediated by invasive apple snails in Jiangsu Province of China”

We thank you very much for the opportunity to revise and resubmit our manuscript and the peer reviewers for taking the time to review the manuscript and for their positive feedback. As detailed in the response to reviewers, we have provided revisions with consideration of each of the reviewers’ suggestions under the track changes mode in the manuscript.

We hope that you will consider our manuscript further.

Yours sincerely,

Yaobao Liu, PhD.

Associate Professor, Jiangsu Institute of Parasitic Diseases (JIPD)

Add: Meiyuan Yangxiang 117, Wuxi, Jiangsu, 214064, P.R.China

Tel: +86-510-6878 1029; Fax: +86-510-8551 0263

Email: yaobao0721@163.com

**Responses to editors**

***1) Please ensure that the CRediT author contributions listed for every co-author are completed accurately and in full.***

***At this stage, the following Authors/Authors require contributions: Qiang Zhang, Fanzhen Mao, Bixian Ni, Jiayao Zhang, Feng Tang, Xinyi Gong, and Yaobao Liu. Please ensure that the full contributions of each author are acknowledged in the "Add/Edit/Remove Authors" section of our submission form.***

***The list of CRediT author contributions may be found here: https://journals.plos.org/plosntds/s/authorship#loc-author-contributions***

**Response**:

We sincerely appreciate the editor’s careful review and valuable feedback. In response to your comment regarding the CRediT author contributions, we have now carefully reviewed and updated the contributions for all authors in the “Add/Edit/Remove Authors” section of the submission system, ensuring that each co-author’s role is accurately and fully described.

***2) We notice that your supplementary Table is included in the manuscript file. Please remove it from the main file of the manuscript as it should be uploaded separately with the file type 'Supporting Information'.***

**Response**:

Thank you for pointing this out. We have carefully followed your instruction. The supplementary table (S1 Table) has now been removed from the main manuscript file and will be uploaded separately during the submission process.

***3) Please amend your detailed Financial Disclosure statement. This is published with the article. It must therefore be completed in full sentences and contain the exact wording you wish to be published.***

***1) State the initials, alongside each funding source, of each author to receive each grant. For example: "This work was supported by the National Institutes of Health (####### to AM; ###### to CJ) and the National Science Foundation (###### to AM)."***

***2) State what role the funders took in the study. If the funders had no role in your study, please state: "The funders had no role in study design, data collection and analysis, decision to publish, or preparation of the manuscript.".***

**Response**:

We have amended it to be presented in full sentences as required. The exact wording for publication is as follows: "This study was supported by the Jiangsu Province Capability Improvement Project through Science, Technology, and Education (No. ZDXYS202207) and Jiangsu Provincial Preventive Medicine Association Research Program (Y12023051). The funders had no role in the conceptualization, design, data collection, analysis, decision to publish, or preparation of the manuscript. " This statement has been updated in the submission system.

**Responses to reviewers**

*Note, line number given in our responses refer to the clean manuscript (i.e., without tracks or track changes version with simple markup viewing)

**Reviewer #1:**

***1.Line 51: Add "health" to the phrase “gaps in public health practices.”***

**Response**:

We thank the reviewer for this helpful suggestion. The word "health" has been added as recommended. The sentence now reads: “…gaps in public health practices.” (Please see Line 52 in the Revised Manuscript with Track Changes).

***2.Line 53: Refine the phrase “We also found that older and less-educated residents had lower awareness and poorer preventive habits.” I suggest "We also observed that older individuals and those with lower levels of education demonstrated lower awareness and exhibited less effective preventive habits." This revision maintains neutrality and avoids potentially stigmatizing language.***

**Response**:

We sincerely thank the reviewer for this valuable suggestion. We agree that the proposed revision enhances the neutrality and academic tone of the statement. We have adopted the suggested sentence verbatim in the revised manuscript. The updated text now reads: "We also observed that older individuals and those with lower levels of education demonstrated lower awareness and exhibited less effective preventive habits." (Please see Line 53-55 in the Revised Manuscript with Track Changes).

***3.Line 67: The role of potable water in the transmission of A. cantonensis remains under investigation, as there is currently no conclusive evidence linking it to disease transmission. In the case of fruits and vegetables (primarily vegetables), contamination may result from contact with gastropod body parts, though this too remains under scrutiny.***

**Response**:

We thank the reviewer for this crucial clarification regarding the evidence for transmission routes. We fully agree that the role of potable water and the significance of produce contamination require more definitive evidence and are areas of ongoing research. Following this comment, we have revised the relevant sentences in our manuscript to reflect this more cautious and accurate scientific consensus. The text now emphasizes that these are potential but not yet conclusively proven pathways. The revised sentence now reads: "Human infection occurs primarily through the intentional or accidental ingestion of third-stage larvae (L3) present in infected intermediate hosts or paratenic hosts. A potential, though less documented, route involves the ingestion of L3 larvae that may contaminate vegetables, possibly via contact with slugs or small snails, or, more theoretically, in water" (Please see Line 68-72 in the Revised Manuscript with Track Changes).

***4.Line 73: For a more comprehensive overview of the global expansion of A. cantonensis, I recommend including references such as https://pubmed.ncbi.nlm.nih.gov/36343594/ https://pmc.ncbi.nlm.nih.gov/articles/PMC10077014/, as the current references (6 and 8) do not address this issue.***

**Response**:

We sincerely thank the reviewer for this excellent suggestion and for providing these highly relevant and up-to-date references. We agree that incorporating these articles will significantly strengthen the background on the parasite's global spread. We have now cited both recommended references (added as new references 8 and 9, please see Line 78 in the Revised Manuscript with Track Changes) in the introduction to replace and supplement the previous citations, providing a more comprehensive overview of the global expansion of *A. cantonensis*. Please see the updated reference list：

[8] Cowie RH, Ansdell V, Panosian Dunavan C, Rollins RL. Neuroangiostrongyliasis: Global spread of an emerging tropical disease. Am J Trop Med Hyg. 2022;107(6):1166-72.

[9] Paredes-Esquivel C, Foronda P, Dunavan CP, H Cowie R. Neuroangiostrongyliasis: Rat lungworm invades Europe. Am J Trop Med Hyg. 2023;108(4):857.

***5.Line 198: While it is important to separate raw and cooked cutting boards to reduce the risk of cross-contamination, further clarification is needed regarding the relevance of this practice for A. cantonensis transmission. The potential for larvae transmission through cross-contamination in this context may be minimal or uncertain, and this should be addressed in the discussion.***

**Response**:

We thank the reviewer for raising this critical point. We agree that the direct relevance of this practice to A. cantonensis transmission requires more precise clarification. We have revised the discussion to better contextualize this finding. The changes are as follows: "Although a certain number of households separated raw and cooked cutting boards and knives, 40.3% did not. While the direct risk of A. cantonensis transmission via cross-contamination from utensils is likely lower than via direct ingestion of infected hosts, this practice gap reflects a broader potential for general foodborne pathogen transmission. It also highlights a crucial area for health education, as improving fundamental food safety practices can concurrently mitigate risks associated with various parasitic and bacterial diseases." (Please see Lines 278-285 in the Revised Manuscript with Track Changes).

***6.Line 242: The statement ‘ecological absence does not equate to population safety’ requires further clarification. If a pathogen is absent from a region at a given time, the specific risk associated with that pathogen may not exist. The phrase could be misleading unless additional context is provided.***

**Response**:

We thank the reviewer for this correct observation. We have revised the statement to eliminate any potential misunderstanding. The text now more precisely reflects our intended meaning that the current absence does not eliminate future risk: "Although no infected snails were detected, the potential for transmission persists due to widespread vectors and unsafe practices, underscoring that current absence should not be equated with enduring safety." (Please see Lines 247-249 in the Revised Manuscript with Track Changes)

***7.Line 251: “The apparent absence in Jiangsu may due to limited sample size, seasonal sampling, or presently unfavorable local ecological conditions” please provide further information on these important variables.***

**Response**:

We thank the reviewer for this suggestion. We agree that providing more context on these potential factors would strengthen the discussion. We have revised the sentence and added further explanation as follows: “The apparent absence in Jiangsu may be attributable to several factors. Although our sample size provides a preliminary overview, it may be insufficient to detect a low prevalence of infection. Furthermore, our sampling was conducted during a single season (the summer reproductive peak), which might not capture the full dynamic of the parasite's life cycle. Lastly, presently unfavorable local ecological conditions, such as low densities of definitive rodent hosts or climatic factors suboptimal for larval development in snails, could also contribute to the absence of detected infection”. (Please see Lines 256-262 in the Revised Manuscript with Track Changes)

***8.Line 270: this is particularly important given the potential for rapid spread of A. cantonensis, especially through contaminated food sources. However, the reference cited [38] specifically discusses raw snail consumption, not waterborne transmission. Therefore, the claim about water sources should be revisited and, if applicable, supported by more relevant references.***

**Response**:

We thank the reviewer for this comment. We agree with the reviewer's assessment. Please note that this specific sentence and the inaccurate mention of "water sources" have already been addressed. In the current version of the manuscript, the discussion on cross-contamination has been entirely reframed, and the revised paragraph now reads: "Although a certain number of households separated raw and cooked cutting boards and knives, 40.3% did not. While the direct risk of A. cantonensis transmission via cross-contamination from utensils is likely lower than via direct ingestion of infected hosts, this practice gap reflects a broader potential for general foodborne pathogen transmission. It also highlights a crucial area for health education, as improving fundamental food safety practices can concurrently mitigate risks associated with various parasitic and bacterial diseases." (Please see Line 278-285 in the Revised Manuscript with Track Changes).

***9.General Comment: The authors should mention the lack of specific public health guidelines, particularly in newly endemic regions of A. cantonensis. Interestingly, in regions where A. cantonensis is endemic, there is a lack of specific public health guidelines to educate the public on the risks associated with gastropod contact. For example, a recent study by [DOI: 10.1111/zph.13228] highlights the need for such guidelines in endemic areas and mention the absence of such guides.***

**Response**:

We thank the reviewer for this valuable observation. We agree that the lack of specific guidelines is a significant public health concern. In response, we have integrated this point into the existing discussion on health education, and have cited the recommended study (DOI: 10.1111/zph.13228) to support the argument. The revised text now includes the following sentence: "The current lack of specific public health guidelines for A. cantonensis in many at-risk regions, including newly endemic areas [43], further highlights the urgency of developing such tailored, evidence-based interventions to translate knowledge into sustained preventive behaviors." (Please see Line 307-310 in the Revised Manuscript with Track Changes).

Please see the updated reference list：

[43] Jaume-Ramis S, Rivory P, Velázquez IS, Šlapeta J, Paredes-Esquivel C. Neuroangiostrongyliasis infection risk near preschool centres in mallorca, Spain: A pilot micro-epidemiological study. Zoonoses Public Health. 2025;72(6):515-22.

***10. Reference 3: Correction needed regarding author name. "Carlos GT" should be corrected to "Graeff-Teixeira" to reflect the proper citation format.***

**Response**:

We thank the reviewer for pointing out this error in the citation. The author's name has been corrected to "Graeff-Teixeira C" in reference 3, and the reference list has been updated accordingly.

**Reviewer #2:**

***1. KAP should be defined the first time it is used, in the abstract***

**Response**:

We thank the reviewer for this reminder. We have revised the abstract to define the acronym "KAP" upon its first mention. The sentence now reads: "Simultaneously, a validated Knowledge, Attitudes, and Practices (KAP) questionnaire was conducted to 630 randomly selected residents." (Please see Line 27 in the Revised Manuscript with Track Changes)

***2. Why is T1 considered as Supplemental? It should be just Table 1 in the paper and adjust other accordingly.***

**Response**:

We thank the reviewer for this question. The primary reason for presenting this dataset as a supplementary table (S1 Table) is that all results from both the field investigation and market survey were negative. While this "null result" is critically important for context and transparency, we feel that placing the detailed table in the supplementary materials allows the main manuscript to remain focused on the positive findings and detailed analysis of the KAP survey, which is the core novel contribution of the study. Presenting the negative results in a succinct manner within the text, while providing the full data in the supplement, is a common and efficient way to structure the findings. We hope this clarification explains our rationale, and we kindly request to keep the table in its current supplementary format.

***3. L201, big space unneeded***

**Response**:

We thank the reviewer for pointing out this formatting issue. The unnecessary large space has been removed in the revised manuscript.

***4. L199, Why is 78.7% respondents referred to as ‘only’ 78.7%. That is a pretty big number of respondents***

**Response**:

We thank the reviewer for this valuable feedback. We understand the reviewer's point. Our use of "only" was made in a relative sense, as this reporting rate (78.7%) is notably lower than the other positive attitude rates in the same survey. However, we acknowledge that the word "only" can be subjective and may unintentionally undervalue a majority figure. Therefore, we have removed it in the revised manuscript to maintain a neutral and objective tone.

***5. L 238 Indent needed for consistency***

**Response**:

We thank the reviewer for this comment. According to the journal's formatting style, the first paragraph under a heading is not indented, while subsequent paragraphs should be indented for consistency. We have checked the manuscript and confirmed that the formatting at Line 238 now adheres to this rule. Thank you for helping us ensure formatting consistency.

***6. L 272.Again, why is 78.7% considered as a ‘low’ reporting rate, to me seems pretty high.***

**Response**:

We thank the reviewer for this pertinent question. We agree that a 78.7% reporting rate is not low. However, we characterize it as an area for improvement ("low" in a relative sense) than the other positive attitude rates in the same survey.

***7. L290 Remove either ‘such as’ or ‘like’, so don’t need both.***

**Response**:

We thank the reviewer for this suggestion. We have removed the word "like" from the sentence as recommended.

***8. Why wasn’t PCR testing conducted, it’s much more sensitive?***

**Response**:

We sincerely thank the reviewer for raising this critical methodological point. The reviewer is absolutely correct that PCR-based methods offer superior sensitivity for detecting low-level A. cantonensis infections. We acknowledge that relying solely on pepsin-HCl digestion and microscopy is a limitation of our study, as stated in the 'Limitations' section of the Discussion (Lines 318-320): " Third, because all specimens were screened solely by pepsin–HCl digestion and microscopy, infections with very low larval burdens may have been missed". We fully agree with the reviewer, and we have explicitly highlighted the need to employ molecular tools like PCR in future surveys to enhance detection sensitivity in the concluding paragraph of the Discussion: “Meanwhile, deploying molecular tools such as conventional PCR, qPCR or LAMP in future surveys will improve detection sensitivity and allow more precise monitoring of the parasite’s transmission dynamics”. (Lines 326-328). We appreciate this insightful comment, which strengthens the self-critical analysis of our work.

**Reviewer #3:**

**Response**:

We would like to express our sincere thanks to Reviewer for the thorough review and the valuable comments provided directly in the manuscript file. We have carefully addressed each point raised in the Word document using the "Comment" feature. For the convenience of the editor and reviewers, our detailed responses to each specific comment can be found directly within the revised manuscript.
